# Past, Present, and Future of Impulse Buying Research Methods: A Systematic Literature Review

**DOI:** 10.3389/fpsyg.2021.687404

**Published:** 2021-07-01

**Authors:** Marco Mandolfo, Lucio Lamberti

**Affiliations:** Department of Management, Economics, and Industrial Engineering, Politecnico di Milano, Milan, Italy

**Keywords:** impulse buying, impulse purchase, systematic review, consumer behaviour, consumer neuroscience, neuromarketing

## Abstract

Impulse buying (IB) represents a pivotal subject in consumer psychology. A general agreement on its core elements and their relationship is arguably established. So far, however, there has been little discussion about how to assess impulse purchases, leading to a potential divergence of practise from theory and complexities in cross-study comparability. This systematic literature review investigates the research methods and metrics employed in high-quality literature to evaluate impulse shopping behaviours across different environments, including online, offline, and multichannel settings. Following the Preferred Reporting Items for Systematic reviews and Meta-Analyses (PRISMA) criteria, the literature search has been conducted on databases relevant for scientific literature, including Scopus, Web of Science, and ProQuest. Fifty-four articles were included in this systematic review. Findings show the existence of four methods to investigate IB, namely quantitative self-reports, laboratory investigations, fieldwork observations, and qualitative interviews. A comparison of the four methods in terms of fit highlights that self-reports and interviews provide a significant contribution in assessing the cognitive facet of impulse purchasing. Laboratory investigations and fieldwork observation find a better fit with the conative and visceral facets of impulsive buying. Considering the major role of affective charges occurring during impulse shopping, complementary research approaches, and metrics belonging to applied psychophysiology and consumer neuroscience are examined. Three opportunities for future research are discussed, including theory building and refinement, understanding individual differences, and honing behavioural predictions.

## Introduction

Impulse buying (IB) represents an established topic in consumer psychology. Several reviews find common ground in describing it as a multifaceted construct, which includes conative, visceral, and cognitive factors (Xiao and Nicholson, 2013; Amos et al., 2014; Chan et al., 2017; Iyer et al., 2020). From its conative side, IB is conceptualised as an act with no pre-shopping intentions driven by immediate self-fulfilment (Rook, 1987; Rook and Fisher, 1995; Beatty and Ferrell, 1998). Conative expressions of IB also include rapid decision-making and on-the-spot actions (Piron, 1991; Lades, 2014). Concerning its visceral facet, IB involves a compelling psychological urge to purchase and a powerful emotional charge (Rook and Gardner, 1993; Wood, 1998; Baumeister, 2002). Impulse buying further stimulates emotional conflict in the post-purchase due to its hedonic content (Puri, 1996; Dittmar and Drury, 2000). Third, regarding its cognitive aspect, IB favours short-term gains triggered by the urgency to seek immediate gratification. This drive appears to be triggered by alluring desires towards the possession of a product (Rook and Hoch, 1985; Dholakia, 2000) and has been related to fallacious intertemporal decisions, where immediate smaller rewards are favoured on delayed greater rewards.

Impulse buying has been investigated from different perspectives (Verplanken and Sato, 2011) and a general agreement on its core elements is arguably established in the literature (Xiao and Nicholson, 2013). So far, however, there has been little discussion about how to assess impulse purchases. This scant consideration of assessment methods might lead to inconsistencies in research. First, inconsistencies between the theoretical conceptualisation of IB and its actual measurement may lead to a divergence of practise from theory. This misalignment mines theory confirmations or confutations and it tends to increase the distance between academics and practitioners (Kumar, 2017). Second, the absence of empirical standards for IB assessment may hamper cross-study comparability in upcoming research. A lack of a common method may further hinder replicability and lead to fragmentation within the same field of research, which is a documented concern in actual IB research (Xiao and Nicholson, 2013; Chan et al., 2017).

The present work intends to bridge this gap by systematically investigating the methods and metrics employed in consumer research to assess IB across different environments, including online, offline, and multichannel settings. This paper offers the scholarly community a consolidated overview of approaches employed in consumer behaviour research. We discuss past and current methods as well as emerging techniques highlighting their features in the different contexts of use. Our argumentations follow a positivist perspective, positing that the facets of IB might be assessed through measurements. This stance seemingly appears to be shared by the greater majority of authors delving into the topic (Beatty and Ferrell, 1998; Hausman, 2000). Along these lines, our intended contribution is two-fold. First, we advance suggestions concerning the suitability of each research method to different research goals. Through a direct comparison of the research methods employed in IB research, we provide indications about the type of research approach and metrics that might be appropriate depending on the context and the specific facet of IB. Second, we provide suggestions about possible metrics borrowed from parallel fields to complement existing research approaches and set directions for forthcoming work.

The remainder of this paper is structured as follows. First, we describe our review methodology, including literature search, selection, and coding. Next, we present our analysis of the literature. Based on the findings of our review, we discuss the fit with IB research of each approach and consider the existing methodological gaps. Lastly, we advance potential directions for future work.

## Review Methodology

A systematic literature review was conducted to analyse IB assessment methods. This approach was chosen due to the maturity of the topic in the marketing and consumer behaviour literature and to foster replicability of results. The 2020 Preferred Reporting Items for Systematic Reviews and Meta-Analyses (PRISMA) statement was used for this article (Page et al., 2021).

### Eligibility Criteria

The initial phase of the research process required defining the search wordings and research boundaries. To avoid an overly narrow stance, our definition of IB included reminder, planned, suggestion, and pure impulse purchases as described by Stern (1962). Likewise, we adopted an extensive selection of research methods including both qualitative approaches (e.g., focus groups, ethnographic studies) and quantitative approaches (e.g., quantitative self-reports, laboratory investigations). Based on these assumptions, we employed a three-layer query. The first layer of the query scanned for documents containing the term “Impulse buying” and the related declinations (i.e., “impulsive buy,” “impulse buy”) in their title, abstract or keywords. Further synonyms were introduced in line with previous reviews (Xiao and Nicholson, 2013; Chan et al., 2017), including “impulse purchase,” “impulse shopping,” and “impulse consumption.” The second layer searched for keywords linked to measurement and assessment methods (e.g., “determinant,” “measure”) and the related synonyms or plurals. The third layer was intended to exclude studies with psychiatric implications (i.e., investigating the sphere of compulsive behaviours), according to the area of investigation of the present study. Therefore, we excluded forms of “compulsive” buying. Since IB behaviours are generally independent of sociodemographic variables (Amos et al., 2014), no further filter was set concerning the population's characteristics.

### Search Strategy and Selection Process

As the existing IB literature is highly interdisciplinary, we queried different databases, namely ScienceDirect/Scopus, Web of Science, and ProQuest. Additional studies were also located by searching papers referenced in listed articles. All the additional articles were retrieved through snowballing from the sample articles. These included studies cited in the methodology as the seminal studies (e.g., Kacen and Lee, 2002; Mattila and Wirtz, 2008) or research method (e.g., Weinberg and Gottwald, 1982).

The structured query included filter related to subject areas, language, and document type. We selected subject areas linked to business and management fields, psychology, as well as social sciences. The documents included were limited to peer-reviewed research published in English, thus excluding conference proceedings and book chapters. An example of the specific query employed on Scopus is reported in Supplementary Figure 1 among Supplementary Material.

The search phase resulted in the collection of 258 documents. All the gathered documents were successive to the seminal paper introducing the concept of IB in the marketing literature, namely Stern (1962), therefore congruent with the chosen definition of IB. In particular, the selected literature ranged from 1982 to 2020. In the screening phase, duplicates were removed based on abstract screening. A total of 93 articles was removed at this stage.

### Inclusion and Exclusion Criteria

Inclusion and exclusion criteria were applied to ensure sample reliability. Inclusion criteria were employed to include high-quality literature. First, to ensure the selection of high-quality research and the appropriate coverage of a wide selection of academic journals, we narrowed our sample by focusing on high-ranked journals. In line with Morris et al. (2009), we included only papers belonging to 2, 3, and 4-rated journals based on the Chartered Association of Business Schools' 2018 Academic Journal Guide (AJG 2018). Seventy nine research papers were excluded after this stage.

**Figure 1:**
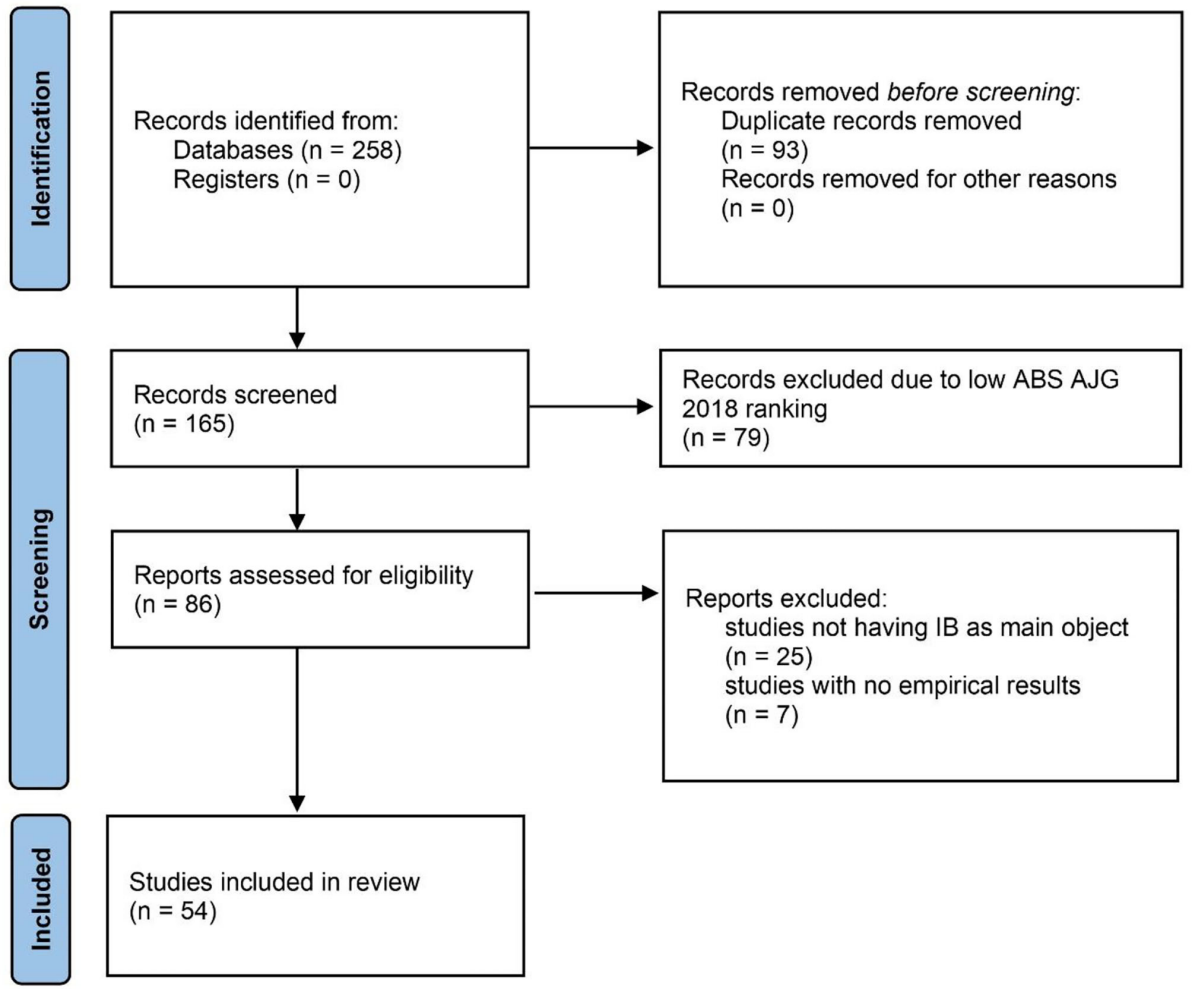
Flow diagram depicting identification and screening process.

Based on the analysis of full texts, we excluded 25 cases where IB was not the main object of the study. Accordingly, we excluded research papers where IB was considered an antecedent of other constructs deemed beyond the present research scope (e.g., store patronage, bidding behaviours). Next, exclusion criteria aimed at removing studies having no empirical results (i.e., cases presenting only theoretical models of IB were excluded). A total of seven documents was excluded after this stage. To minimise the risk of bias, inclusion and exclusion criteria were independently evaluated by the two authors. Overall, 54 documents were deemed relevant at the end of the search process. Figure 1 illustrates the flowchart of the study selection process, which was based on the PRISMA 2020 protocol (Page et al., 2021).

### Information Extraction and Coding

The following step of the review process concerned the categorisation of the gathered literature. To systematically document the cataloguing process, we extracted the following information from each study: (i) research approaches, namely the typology of research to investigate IB (e.g., self-reports, focus groups, laboratory investigations); (ii) metrics to assess or measure IB (e.g., the proportion of items bought on impulse); (iii) data collection technique (e.g., questionnaires, devices for data gathering); (iv) data analysis technique [e.g., structural equation modelling (SEM), econometric models]; (v) sample size; and (vi) typology of IB (i.e., in-store, online, multichannel, generic).

## Results

### Characteristics of the Studies

Despite being a well-established construct in the consumer behaviour literature, the assessment of IB appears to be mainly developed in the last two decades. Beatty and Ferrell (1998) might be deemed the seminal paper for pioneering the application of SEM to multiple hypothesis testing. The last decade saw the starkest development in terms of published research with 31% of the total publications in the period 2010–2015 and 39% from 2016 to 2020. The advent of the internet allowed to broaden the conceptualisation of IB, framing it also in the online environment (Adelaar et al., 2003; Parboteeah et al., 2009). Accordingly, our sample shows not only studies analysing IB in the general context (41%), but a relevant part concerns the online setting (33%). The remaining slice encompasses both studies investigating IB in specific in-store sites (22%) or multichannel realities (4%), where consumers had interactions with both digital tools and brick-and-mortar realities (e.g., Bellini and Aiolfi, 2019).

### Approaches to Investigate Impulse Buying

The large majority of the collected studies (94%) adopted quantitative approaches in assessing IB. Among these, we observed a prevailing line relying on self-reports (63%), laboratory investigations (26%), and fieldwork observations (11%). Alternative qualitative methods prove to be entirely based on face-to-face interviews.

#### Quantitative Self-Reports

Self-reports were often assessed through closed-ended scales evaluating the level of agreement on agree-disagree scales or between semantic items. These scales were regularly adapted from multiple-item scales present in the prior marketing literature (Silvera et al., 2008; Lucas and Koff, 2014) or on established psychometric reference scales (Mehrabian and Russell, 1974; Watson et al., 1988). Alternative approaches to assess IB were found in hypothetical scenarios descriptions investigating the personal evaluations of the respondent on semantic close-ended scales (Chih et al., 2012; Liu et al., 2013).

Impulse Buying appears to be assessed through self-reports either as a personality trait or as a measure of recalled past acts of buying. Impulse buying-related personality traits prove to be entirely assessed through multiple-item scales, though not univocally defined. The case of IB tendency is emblematic. Three different major scales are employed to assess IB tendency, namely the ones developed by Rook and Fisher (1995), Weun et al. (1998), and Verplanken and Herabadi (2001). The 9-item scale developed by Rook and Fisher (1995) appear to be the most frequently used tool (tallied 16 times as significant), while the other two scales are frequently used as well (each tallied 7 times). No relevant difference was spotted in the temporal adoption of any of the three, being all used both in earlier (Beatty and Ferrell, 1998; Kacen and Lee, 2002; Silvera et al., 2008) and recent research (Bellini et al., 2017; Liu et al., 2019; Meng et al., 2019). Content-wise, the scale developed by Verplanken and Herabadi (2001) takes a different angle, discriminating between cognitive and affective facets of the phenomenon and hence proves to be more often adopted in psychology-related studies (Lucas and Koff, 2014, 2017). To the best of our knowledge, no specific study has tackled the issue of comparing the three scales in terms of effectiveness. Notwithstanding, more recent studies contributed to the development of further scales that consider also the conative facet of IB in a cross-cultural setting (Sharma et al., 2014) or assess it as a transient individual state reflecting temporary depletion of self-regulatory resources (Lucas and Koff, 2014). It is relevant to note that other significant personality traits are mostly assessed through established scales adopted in other research fields, including marketing, sociology, and psychology (Das, 2016; Olsen et al., 2016; Meng et al., 2019). This underscores the interdisciplinary interest in assessing IB behaviours.

The investigation of recalled acts of buying is variegated. Direct queries are frequently used to determine the essence of the purchase. These are recorded through multiple-item Likert scales evaluating the unplanned side of IB (e.g., “I ended up spending more money than I originally set out to spend,” and “I bought more than I had planned to buy,” Mattila and Wirtz, 2008, p. 564), its spontaneity (Verhagen and Van Dolen, 2011), or single direct question (i.e., “How often do you buy things on impulse?,” Kacen and Lee, 2002, p. 167). Alternatively, the perceived urge to buy was employed as a proxy of IB behaviours, assessed on multiple-item Likert scales (e.g., “I had the urge to purchase items other than or in addition to my specific shopping goal,” Parboteeah et al., 2009, p. 67). A further method based on self-reports consisted in the employment of imaginary shopping situations. These require the subjects to deviate from any personal shopping goal and project themselves into a hypothetical shopping scenario suggesting a purchasing choice to a fictional third character (e.g., “Mary is a 21-year-old college student with a part-time job,” Rook and Fisher, 1995, p. 308). Table 1 summarises IB metrics, their description with the related primal reference scale, and reference studies.

**Table 1:** IB metrics assessed through quantitative self-reports.

	**IB metric**	**Description**	**Primal reference scale**	**Reference study**
IB-related personality traits	Impulse buying tendency	Individual disposition to buy on impulse	Rook and Fisher, 1995	Kacen and Lee, 2002; Jones et al., 2003; Vohs and Faber, 2007; Parboteeah et al., 2009; George and Yaoyuneyong, 2010; Sun and Wu, 2011; Hubert et al., 2013; Liu et al., 2013; Serfas et al., 2014; Chen and Wang, 2016; Das, 2016; Olsen et al., 2016; Chung et al., 2017; Khachatryan et al., 2018; De Vries and Fennis, 2019; Meng et al., 2019
			Weun et al., 1998	Beatty and Ferrell, 1998; Kacen and Lee, 2002; Adelaar et al., 2003; Chih et al., 2012; Mohan et al., 2013; Shukla and Banerjee, 2014; Bellini et al., 2017
			Verplanken and Herabadi, 2001	Silvera et al., 2008; Lucas and Koff, 2014, 2017; Dhaundiyal and Coughlan, 2016; Olsen et al., 2016; Bossuyt et al., 2017; Liu et al., 2019
	Overall consumer impulsiveness	Cross-cultural disposition to purchase impulsively	Sharma et al., 2014	Sharma et al., 2014
			Mittal et al., 2016	Mittal et al., 2016
	Impulsive behaviour	Individual disposition to act on urges with little deliberation or evaluation of consequence	Puri, 1996	Sharma et al., 2010; Hubert et al., 2013, 2018
			Whiteside and Lynam, 2001; Cyders et al., 2007	Lucas and Koff, 2014
Act of buying	Impulse buying behaviour	Expression of impulsive buying behaviour during the act of purchase	Mattila and Wirtz, 2008	Mattila and Wirtz, 2008; Badgaiyan et al., 2017; Meng et al., 2019
			Verhagen and Van Dolen, 2011	Verhagen and Van Dolen, 2011; Chung et al., 2017
			Kacen and Lee, 2002	Kacen and Lee, 2002; Lee and Kacen, 2008
			Yoon, 2013	Yoon, 2013
			Badgaiyan et al., 2017	Badgaiyan et al., 2017
	Recent impulse buying behaviour	Individual transient impulse buying behaviour	Lucas and Koff, 2014	Lucas and Koff, 2014
	Impulsive purchase decision	Willingness of a customer to perform the act of purchase	Rook and Fisher, 1995	Parboteeah et al., 2009; Chih et al., 2012; Chen and Wang, 2016
	Felt urge to buy impulsively	State of desire experienced upon encountering a purchasing object	Luo, 2005	Luo, 2005; Chen and Wang, 2016; Meng et al., 2019
			Parboteeah et al., 2009	Parboteeah et al., 2009; Liu et al., 2013; Chen et al., 2019
			Adelaar et al., 2003	Adelaar et al., 2003; Martínez-López et al., 2020

Results showed that closed-ended surveys represent the most adopted data collection instrument. Nevertheless, discrepant strategies for data collection were observed. A consistent part of the studies employed either directly handed-out surveys or online surveys (Sun and Wu, 2011; Liu et al., 2013). Other study protocols favoured mall-intercept methods involving customers in stores before and after their shopping experience (Beatty and Ferrell, 1998; Mohan et al., 2013; Bellini et al., 2017). Further pieces of evidence point out the use of consumer shopping diaries combined with questionnaires to restrain from the presence of the interviewer (Jones et al., 2003). Among the studies employing self-reports, the respondent sample proved variable in size, averaging roughly 400 respondents. The greater part (71%) involved between 200 and 600 respondents, while larger (11%) and smaller (18%) samples were observed as well. Given the multi-faceted nature of IB, larger samples were common practise to meet the minimum sample size to perform SEM in the subsequent data analysis. Data analyses based on self-reports were frequently carried out through SEM (46% of total quantitative studies), whereas econometric models or inferential analyses accounted for the remaining part.

#### Laboratory Investigations

Our findings point out a variegated pool of laboratory investigations. These are intended to induce and observe IB in an artificial and controlled environment. Notable laboratory investigations are found both in online IB explorations (Adelaar et al., 2003; Parboteeah et al., 2009) and in general offline IB behaviours (Weinberg and Gottwald, 1982; Vohs and Faber, 2007). Such experimental tests follow experimental designs focused on either eliciting actual IB behaviours against controlled responses or manipulating contextual variables to assess their impact on purchasing intentions. For instance, Weinberg and Gottwald (1982) recreated a situation where subjects decided spontaneously on purchasing real products. Likewise, Bossuyt et al. (2017) conceived a real shopping task where participants, after receiving a budget from a predetermined lottery, faced either a category of products with high or low hedonic components (i.e., sweet snacks or rice and pasta). Differently, Adelaar et al. (2003) tested three distinct digital media characterised by different cues to assess their impact on urges to buy impulsively.

Different experimental investigations analyse the behavioural expression of IB. A prominent experimental design was employed by Vohs and Faber (2007) to explore the effect of self-regulatory resources depletion. Through three experiments, the authors initially depleted individuals' cognitive resources through attention-control tasks. Afterwards, participants received a monetary endowment and faced the decision to either pocket the money or use a part to perform an immediate purchase. Willingness to pay, the amount spent, and the purchased quantity were employed as dependent variables. A second notable example is provided by De Vries and Fennis (2019) who examine through a multiple-study how local brands may induce low-level construals, promoting IB behaviours. Through an online study, subjects faced an actual website selling actual products characterised by different brand positioning, which could be voluntarily purchased exchanging the monetary endowment related to their participation.

A few studies adopted a radically distinct approach, proposing to assess IB, and the related visceral activations from the physiological and behavioural responses of the participant. Early studies conceptualised the possibility to assess the perceived arousal during IB processes through electrodermal activations (Weinberg and Gottwald, 1982), whereas recent studies tested its methodological applicability (Bossuyt et al., 2017). Other research studies employed functional Magnetic Resonance Imaging to measure sub-cortical neural activations in conjunction with stimulating product packaging to delve into the underlying visceral processes characterising an impulse purchase (Hubert et al., 2013, 2018). Further studies assessed the affective state related to the act of impulse purchase from the ocular patterns and pupil dilation of the user, as a proxy of physiological arousal (Huang and Kuo, 2012; Serfas et al., 2014; Khachatryan et al., 2018). Further evidence of the manifestation of arousal through facial mimic was provided by Weinberg and Gottwald (1982) who monitored the facial expressions of buyers and non-buyers during a potential act of purchasing. Table 2 shows the observed IB metrics assessed in the laboratory setting with the related indicators and reference studies.

**Table 2:** IB metrics assessed through laboratory investigations.

	**IB metric**	**Description**	**Indicator**	**Reference study**
Act of buying	Impulse buying behaviour	Expression of impulsive buying behaviour during the act of purchase	Real unplanned purchase	Vohs and Faber, 2007; De Vries and Fennis, 2019
			Experimental product category chosen	Bossuyt et al., 2017
	Physiological and behavioural response	Individual reactions encompassing individual's central or peripheral nervous system activity, instinctive non-verbal actions or behavioural exteriorisation	Neural cortical and sub-cortical activations	Hubert et al., 2013, 2018
			Decision time	Huang and Kuo, 2012
			Information ocular search patterns	Huang and Kuo, 2012
			Pupil dilation	Serfas et al., 2014
			Ocular fixations count	Khachatryan et al., 2018
			Facial expressions	Weinberg and Gottwald, 1982
			Electrodermal activations	Bossuyt et al., 2017

These laboratory investigations remarkably share some common features. First, participants usually lack prior awareness of the purchasing possibility. The creation of a setting devoid of anticipatory cues pointing to a purchasing context recalls the unplanned nature of an impulsive purchase characterised by no pre-shopping intentions (Stern, 1962; Rook, 1987). Second, these experimental designs grant the possibility of performing a deliberate purchase. The spontaneous purchasing decision echoes the characteristic of IB behaviours of being on-the-spot decisions (Piron, 1991). Third, the conceived designs provide a monetary endowment framed as compensation for the participation. Giving a monetary sum that can be instantly disbursed instead of pocketed results to trigger the conative facet of IB, triggering urgency to seek immediate gratification (Rook and Hoch, 1985; Hoch and Loewenstein, 1991).

#### Fieldwork Observations

The third approach involves the assessment of IB in the actual shopping context. Research studies following this approach investigate the outcome of actual purchases in a naturalistic setting, commonly a shopping venue. For instance, Beatty and Ferrell (1998) adopted mall-intercept surveys pre- and post-shopping experience, comparing the actual purchases with initially planned ones. The method implies a first contact, where the subjects are asked to identify their shopping plans, which are then compared with the actual purchases after the shopping trip to discriminate the nature of the buying process. Similar methods investigated the proportion of unplanned purchased items (Mohan et al., 2013; Bellini et al., 2017) or the number of actual impulse purchases gathered from individual shopping diaries (Jones et al., 2003). A further notable setting is found in the application of Virtual Reality. Schnack et al. (2019) recreated an immersive virtual convenience store where subjects had to perform actual purchases while behavioural metrics were tracked. In Table 3 we report the spotted IB metrics assessed through fieldwork observations with the related reference studies.

**Table 3:** IB metrics assessed through fieldwork observations.

	**IB metric**	**Description**	**Indicator**	**Reference study**
Act of buying	Impulse buying behaviour	Expression of impulsive buying behaviour during the act of purchase	Proportion of items bought on impulse	Mohan et al., 2013; Bellini et al., 2017; Bellini and Aiolfi, 2019
			Categorical variable ranging from 1 (no/planned purchase) to 3 (impulse purchase)	Beatty and Ferrell, 1998; Sharma et al., 2010
			Number of actual impulse purchases over a given time	Jones et al., 2003; Parguel et al., 2017
			Number of actual purchases in an immersive virtual store	Schnack et al., 2019

Fieldwork observation follows a naturalistic stance of investigation, embracing principles of ethnographic studies, namely non-participant observation of the purchasing process. Accordingly, the assessment of IB behaviours is reflected in actual purchases, commonly without any manipulation of environmental variables. It is worthwhile to note that all the empirical observations were paired with post-experience surveys. These are often aimed at assessing further variables such as personality traits or perceived affective states (e.g., Beatty and Ferrell, 1998; Mohan et al., 2013).

#### Qualitative Interviews

The fourth approach is represented by interviews. Our results show that interviews delving into IB follow a semi-structured format resembling an open and naturalistic conversation with a single respondent (e.g., Dittmar et al., 1995). The technique involves a thematic text analysis, namely an interpretation and a further categorisation of verbal information into theme-based patterns related to the understanding of meanings and motivations associated with IB episodes (Dittmar and Drury, 2000). Alternatively, other authors favour the adoption of friendship pair interviews or self-scripts (Bayley and Nancarrow, 1998). Friendship pair interviews embody a subset of interviewing techniques where the respondents are recruited as close acquaintances to explore attitudes, motivations, and behaviours in a spontaneous manner. Self-scripts require the respondents to put in writing their experience in a third-person view, thus releasing self-censorship (Bayley and Nancarrow, 1998).

Our results show that qualitative interviews provide insights into broader factors related to individual meanings associated with the phenomenon. For instance, from the interviewee's transcripts it was possible to extrapolate factors such as post-purchase regret or the connexion between unplanned and impulsive purchases (Dittmar and Drury, 2000). Furthermore, from a combination of qualitative interviews with quantitative observations, Dittmar et al. (1995) provide evidence for the relationship between identity-relevant products and IB. As concerns sample size, our results show that interviews relied on smaller samples, ranging from 30 to 46 subjects, thus favouring depth over breadth of analysis.

## Discussion

This systematic review underscores that the methods employed to assess IB in consumer behaviour research are various. Therefore, we deem appropriate to set out our discussion with a comparison of the different approaches highlighting their fit to the characteristics of IB. Directions for future research are discussed in the following.

### Research Approaches Comparison

The four catalogued approaches imply different specificities in terms of research design, skills and knowledge, data collection methods, context of use, and costs (Cassar and Friedman, 2004; Given, 2008; Nardi, 2018). Each approach also allows investigating IB from a different perspective. For instance, laboratory investigations allow tracking the temporal progression of a purchasing action from the first encounter with a product to the buying decision. Survey research, on the other hand, may discount the sequence of behavioural actions but facilitate delving into the buyer's rationalisation of the purchasing act. On this premise, we claim that the four research approaches might find a proper fit in assessing a specific facet of IB, namely its cognitive, conative, or visceral side.

Self-reports and qualitative interviews provide a significant contribution in assessing the cognitive externalisations of IB. This cognitive facet includes the understanding and interpretation of the phenomenon. Answers provided through self-reports require that respondents must determine their response through introspection. Accordingly, this process focuses on the rationalisation of behaviour (Nardi, 2018). Self-reports and interviews may prove to be effective in investigating the buyer's justification of determinants and consequences of IB. These include the perception of urges to buy, pre-shopping intentions, representations of future states, or post-purchase dispositions. In other words, self-reports and interviews provide measures of information that cannot be measured directly through observation but demand a narrative framing from the buyer. Furthermore, self-reports and interviews are generally flexible to the context of use, namely they do not require the use of specific measurement instrumentation. Therefore, these approaches can be applied to most research settings that involve an interaction with the participant.

Despite their potential in the assessment of latent constructs, quantitative self-reports and interviews involve an intrinsic risk of responses' reliability. Biases in responses triggered by social desirability, acquiescence in the respondent, or alteration in response recalling, play a central role in IB research. For instance, since IB appears to be often linked to unfavourable consequences or pure irrationality, respondents might be prone to answer in a socially desirable manner (Parboteeah et al., 2009). Researchers need to consider appropriately their research design when questioning IB. To mitigate the biassing effects, specific strategies should be considered. These include the use of social desirability scales, forced-choice items, or the introduction of disincentives to misreport (Nederhof, 1985; Fischer and Fick, 1993).

Behavioural responses are linked to the second facet of IB, namely its conative side. Laboratory investigations and fieldwork observations arguably represent suited research approaches to assess purchasing actions with reference to their rapidity or spontaneity. These approaches imply a direct observation of the purchasing action, thus allowing the monitoring of non-verbal behaviours and their reaction time. The tracking of instinctive behavioural responses paves the way for the assessment of distinctive typologies of impulsivity, including behavioural and process impulsivity. Behavioural impulsivity is manifested as the propensity to make spontaneous purchasing decisions (Koufaris, 2002). Impulsive purchasing behaviours may be evaluated through the amount spent impulsively or the willingness to restrain an impulse (e.g., Vohs and Faber, 2007). Process impulsivity, on the other hand, is manifested as a bounded will to perform a comprehensive evaluation of the product attributes (Pieters and Wedel, 2007). To assess this aspect, information search patterns or behavioural interactions with the purchasing environment may be tracked.

Laboratory investigations by their nature promote significant internal validity, given the possibility of controlling most of the nuisance variables. On the other hand, fieldwork research does not involve any manipulation of environmental variables, thus entailing higher external validity. Accordingly, the choice of the research approach should be guided by the research objective and variables at play. When focussing on the effect of a single modulating variable, investigations carried out in a controlled setting may be favoured. On the contrary, fieldwork observation tends to be better suited to investigating actual scenarios involving a set of complex interactions.

Lastly, with regards to the assessment of the visceral facet of IB, laboratory investigations and fieldwork observation may provide the greatest contribution. Since the visceral facet of IB is related to a sudden emotional charge, the observation of the buyers' responses in real-time is central. The assessment of these sudden alterations in the individual's affective state might be performed through non-verbal responses such as facial expressions and proxemics (e.g., Weinberg and Gottwald, 1982) or through physiological responses (e.g., Bossuyt et al., 2017). The monitoring of visceral responses allows a direct measurement of psychological impulsivity, intended as a sudden feeling, desire, or urge to buy (Rook, 1987; Piron, 1991).

Positing a fit between the four research methods and the three facets of IB, the results of our systematic review highlight two notable patterns. First, we spot a tendency to focus extensively on self-reported measures. The majority of previous studies proves to be limited to surveys aimed at inquiring individual thoughts and contextual evaluations, thus potentially oversighting the implicit mechanisms driving the buying act. Second, we underscore a lack of real-time assessment of IB. Despite little evidence of alternative research methods, common practises in IB research tend to collect data with a time lag between the purchasing act and its measurement. Both elements emphasise that past and current research have focused substantially on the cognitive facet of IB. Instead, the assessment of IB through its conative and visceral facet appears to be still in its infancy. This reliance on cognitive assessments of IB influences both its theoretical understanding and the related practical applications. From the theoretical perspective, this approach may cause a misalignment between the conceptualisation of IB and its actual assessment. Indeed, measurement practises represent a substantial basis for empirically testing theoretical hypotheses and assess consistent knowledge for future research. On the practitioners' side, the issues might affect the reliability of the information which is used as the foundation to deploy marketing actions.

### Directions for Future Work

The present review encourages the adoption of a broader perspective in the assessment of IB. We argue that future research should not exclusively gravitate around the cognitive side of IB, but rather it should encompass methods analysing the visceral and conative facets of IB. To overcome the two issues identified (i.e., over-reliance on self-reports and lack of real-time assessment), we posit that current IB research may be complemented with a broader set of investigation methods. We specifically refer to the use of applied psychophysiology tools, namely the analysis of physiological and behavioural responses to delve into IB behaviours.

Physiological analyses are intended to assess the individual's reactions based on responses related to either the central or peripheral nervous system activity. We expect that IB research may be a fertile ground for the application of physiological analyses, considering the major role of affective charges as well as the rapidity and the powerful behavioural drives that characterise IB. For instance, research might greatly benefit from the assessment of physiological activations in conjunction with atmospheric triggers (e.g., ambient music or lighting condition) to examine the impact of cues that prompt IB. Behavioural analyses represent a parallel approach. We argue that investigations may be broadened by analysing metrics such as the decisional time, gaze behaviour, or vocal expressions during the act of purchase. For instance, the amount of information processed before the purchase might be assessed through decisional time or ocular search patterns. Correlates of impulsivity can be also investigated through behavioural tasks. These include tasks to measure risk propensity or impulse control, such as the Balloon Analogue Risk Task or the Cued Go No-Go Task (Lauriola et al., 2014).

Our argumentation is aligned with previous methodological observations positing that research may benefit from the adoption of complementary methods to self-reports (Scherbaum and Meade, 2013; Bell et al., 2018). Drawing upon this argument, we identify three opportunities for future IB research: theory building and refinement, understanding the role of individual differences, and honing behavioural predictions.

First, theory building and refinement may stem from the understanding of the boundary conditions of the current theories. For instance, extant research often associates affect with a trigger or a state concurrent to the impulsive purchase (Amos et al., 2014). However, the construct of affect is often broadly outlined. Analysing the physiological responses before, during, and after the moment of purchase may shed light on the nature of affective states involved during IB. Psychophysiological tools also allow discerning the temporal evolution of the externalisation of affect and assess how it influences the purchasing process. From this perspective, forthcoming research may find fertile ground in the investigation of the latency time in decision-making, or the role played by cool-down phases. Comparing visceral responses and subjective rationalisations may further clarify their relationships. In particular, future research can benefit not only from the positive correlations between visceral and cognitive responses but from their tensions. Since IB is often characterised by an emotional conflict, insights are expected to emerge from the analysis of discordances between reflective and impulsive responses. Along these lines, prospective investigations may re-examine the categorisations of IB, thus sharpening the classification earlier advanced by Stern (1962).

Second, applied psychophysiology tools may help to understand the role of individual differences, namely why some individuals are more prone to impulse purchases than others. The application of these tools may shed light on the neural structures and physiological responses involved in impulse purchases. They also support the investigation of state-dependent variability. Namely, understanding how different contingent physiological states drive some individuals to purchase impulsively. Along these lines, future research might investigate how temporary states (e.g., stress or fatigue) trigger impulse purchases.

Lastly, applied psychophysiology tools enable gathering additional data useful for improving behavioural predictions. Practitioners may specifically benefit from the enrichment of current multifactorial models (e.g., Prashar et al., 2015) combining self-reports and psychophysiological measures. Predicting the occurrence of online IB has notable marketing implications. For instance, marketers might increase their knowledge about the effectiveness of marketing stimuli such as product placements or promotional campaigns. Moreover, given the relation between IB and product return behaviour (Kang and Johnson, 2009), future research might focus on predicting the occurrence of product returns as a consequence of impulse purchases. The adoption of multimodal research approaches may further shed light on the weight of each facet of IB and highlighting the role of individual and situational factors.

## Limitations

Our results might be subject to certain limitations related to the literature selection process. The systematic search process carried out is dependent on our main query. In our search approach we scanned for documents published in renowned academic journals, hence we intentionally excluded conference papers and books. In doing so we cannot exclude having omitted novel experimental literature and monographs studies. Furthermore, with the decision to exclude studies with psychiatric implications such as compulsive buying behaviours, we have potentially neglected a part of the literature adopting psychophysiological tools. Drawing from related clinical literature, the research approaches based on physiological responses might be enriched to define biomarkers or behavioural indicators related to IB.

## Data Availability Statement

The original contributions presented in the study are included in the article/Supplementary Material, further inquiries can be directed to the corresponding author/s.

## Author Contributions

MM and LL conceived and structured the study. MM wrote the first draft and each section of the manuscript. All authors contributed to the article, final revision, and approved the submitted version.

## Conflict of Interest

The authors declare that the research was conducted in the absence of any commercial or financial relationships that could be construed as a potential conflict of interest.
