# Supplementary material for: Past, Present, and Future of Impulse Buying Research Methods: A Systematic Literature Review
Source: Front Psychol. 2021 Jul 1;12:687404. doi: 10.3389/fpsyg.2021.687404 (PMC8282203; doi:10.3389/fpsyg.2021.687404)
Supplement: Supplementary file 1 [file Table_1.DOCX]

Supplementary Material

**Figure S1**. Original query structure, as employed on Scopus

|  | **Original query** |
| --- | --- |
| 1 layer | **TITLE-ABS-KEY ( ("impuls* buy*" OR "impuls* purchas*" OR "impuls* shop*" OR "impuls* consumption***")** |
| 2 layer | **AND  ("determinant*"  OR  "precursor*"  OR  "antecedent*" OR "predictor*" OR "moderator*" OR "measure*" OR "indicator*" OR "index*") )** |
| 3 layer | **AND NOT  ( TITLE-ABS-KEY ("compuls*") )** |
| Filer 1 | **AND  ( LIMIT-TO ( SUBJAREA ,  "BUSI" )**  **OR  LIMIT-TO ( SUBJAREA ,  "SOCI" )**  **OR  LIMIT-TO ( SUBJAREA ,  "ECON" )**  **OR  LIMIT-TO ( SUBJAREA ,  "PSYC" )**  **OR  LIMIT-TO ( SUBJAREA ,  "DECI" ) )** |
| Filter 2 | **AND ( LIMIT-TO ( LANGUAGE ,  "English" )** |
| Filter 3 | AND  ( LIMIT-TO ( DOCTYPE ,  "**ar**" ) OR  LIMIT-TO ( DOCTYPE ,  "**re**" ) ) |
